# Experience of naturally occurring peer support among people using psychiatric day-care in China: an interpretative phenomenology approach

**DOI:** 10.3389/fpsyt.2024.1349778

**Published:** 2024-04-24

**Authors:** Yijun Fan, Xiao Liu, Conghong Li

**Affiliations:** ^1^ School of Nursing, Shanghai Jiao Tong University, Shanghai, China; ^2^ Department of Nursing, Shanghai Mental Health Center, Shanghai, China

**Keywords:** peer support, mental rehabilitation, community mental health services, interpretative phenomenological analysis, China

## Abstract

**Introduction:**

China presently does not have an established peer support system, and the efforts of peer support workers are not acknowledged in the context of the growing global trend of community-based mental rehabilitation. This study aims to examine the first-hand experiences of persons who participate in psychiatric day-care facilities in Shanghai, China, and receive support from their peers. The goal is to gain a better understanding of how these informal peer support programs function and provide valuable knowledge for the establishment of more structured peer support programs that align with Chinese social culture.

**Methods:**

A total of 14 participants attending psychiatric day-care centres were selected for face-to-face semi-structured in-depth interviews using snowball sampling. The interviews took place between July 2021 and February 2022. The text data of the interview were acquired through transcription and then augmented using the interview scripts and additional resources. The data were analysed using interpretive phenomenological analysis until the themes reached saturation.

**Results:**

A total of three overarching themes and eight corresponding sub-themes were produced: 1. reconstructing a social network: an ordinary interpersonal connection, becoming and conducting oneself, proceeding the process of adaptation in the company of peers; 2. balance and multiple roles within the relationship: selective self-exposure, managing proximity and distance; 3. sense of meaning and sense of community: supporting others while empowering oneself, love as expanding consciousness, advocating for the notion of group identity.

**Discussion:**

This study is the initial examination of the contact and naturally occurring peer support that takes place among individuals in psychiatric day-care centres in China. The study’s findings revealed that participants interact with others who have undergone similar conditions in the day-care setting, enabling them to rebuild an important social network. It is crucial to consider the possible benefits of peer support, assess the obstacles, and facilitate the personal recovery of individuals with mental disorders using the theory of recovery.

## 1 Introduction

Peer support among individuals with mental illness entails a mutually beneficial system of exchanging assistance, where individuals who have personally encountered and triumphed over the difficulties of mental illness can offer hope, companionship, and encouragement to others who are now facing similar circumstances (1, 2). Peer support has been acknowledged as one of the ten fundamental elements of the rehabilitation process by Substance Abuse and Mental Health Services Administration (SAMHSA). It is comprised of three distinct categories: mutual support groups, peer-run services, and peer-led providers in clinical and rehabilitation settings (3). Peer-led support is a formal form of help, and for many years, numerous countries have developed uniform guidelines and training programs (4–6).

Peer support has the potential to mitigate symptoms, enhance hopefulness and overall quality of life in the long run (7). Recent meta-analyses and reviews of randomised trials investigating peer support for individuals with mental illness provide evidence that peer support interventions can effectively reduce symptoms of clinical mental illness, enhance personal recovery, particularly in terms of hope, and serve as a cost-effective and easily implementable intervention that may complement professional treatment (8). Various peer support worker (PSW) positions have been established and standardised globally, including duties such as peer companions, peer advocates, consumer case managers, peer specialists, peer counsellors, and others (9, 10).

Nevertheless, a significant portion of the literature has concentrated on formal peer support programmes administered by PSWs (11, 12). Insufficient emphasis and consideration have been placed on informal peer-to-peer interactions that extend across an individual’s community, environment, personal life, and social network (3). In China, the primary connection between individuals with mental challenges and others falls on their parents (13). Conducting studies on the naturally occurring peer interactions and communication among individuals with mental illness in community settings is crucial, particularly in countries or regions without established formal peer support services. According to our existing clinical observation, people using psychiatric day-care centres in China are more isolated from society, and interactions among peers play a vital role in their lives and recovery from illness. Psychiatric day-care centres are a great source of socialisation for them, but it is not clear what kind of support they receive, which has led us to conceive this study.

China currently lacks a formalised peer support system and does not recognise the work of PSWs. In recent years, there has been a significant increase in the proportion of individuals with mental problems in China who are seeking treatment; and efforts have been made to enhance the community-based rehabilitation approach. Community mental health practise primarily emphasises clinical recovery and does not sufficiently prioritise personal recovery-oriented practises, such as peer support services (13). In highly developed urban areas such as Shanghai, the government has implemented dedicated day care facilities, such as ‘Sunshine Soul Park’, to cater to those with profound mental problems and assist them in their rehabilitation process. These centres are equipped with social workers and other personnel for administration, and doctors make regular visits to offer guidance (14).

At the moment, while experimental studies on peer support programmes implemented in clinical settings have been conducted in mainland China (15, 16), there is a scarcity of research specifically examining peer support among individuals utilising community day care. Our understanding of the dynamics of contact and communication among individuals in day care centres, as well as the influence of peer connections on the process of rehabilitation, remains limited.

Hence, the objective of this study was to investigate lived experience of naturally occurring peer support among individuals utilizing psychiatric day-care services in Shanghai, China. The results of this study will offer valuable insights for the development of formal peer support programs within the context of Chinese social culture.

## 2 Materials and methods

### 2.1 Study design and setting

We selected ‘Sunshine Soul Park’, a day-care centre operated by the China Disabled Persons Federation (a semi-governmental agency) and the Ministry of Civil Affairs in Shanghai, as our leading investigation site. This network of social welfare institutions offers community-based rehabilitation services for individuals with convalescent mental illnesses (14), aiming to facilitate their recovery and alleviate the burden on their families. Patients become eligible to apply for a mental disability certificate one year after discharge from a psychiatric hospital, which grants them access to ‘Sunshine Soul Park’ after an additional year. The facility provides weekday programs including day-care services, psychological counselling, occupation therapy, and various artistic activities such as crafts, singing, and calligraphy. Despite its extensive coverage across the city, ‘Sunshine Soul Park’ is overall understaffed.

### 2.2 Participants

We employed snowball sampling to recruit participants based on their recovery stasis and length of hospitalisation. The sample size was sufficient to achieve data saturation, where no new themes or sub-themes emerged from the interview data. Additionally, it was small enough to facilitate a comprehensive analysis of each participant’s experiences using interpretative phenomenological analysis (IPA).

Criteria for participation included the following: aged between 18 years and 65 years; diagnosed with schizophrenia, bipolar disorder and other psychiatric disorders that meet the ICD-10 diagnostic criteria and currently in a state of recovery; having undergone day-care centre service for at least twelve months with most acute psychiatric symptoms resolved and significant self-awareness regained; having complete reading and comprehension skills with good listening and expression skills; having the capacity to provide informed consent. Exclusion criteria concerned individuals diagnosed with severe physical disabilities and those at risk of distress due to participation, such as acute psychotic symptoms that substantially impact functioning or high risk of self-harm or harm to others.

### 2.3 Ethical considerations

We first contacted the relative managers of psychiatric day-care centres to assess the suitability of potential participants for the study. All participants involved in the study were informed in detail about the research purpose and procedures in the presence of the day-care centre managers. Participants were encouraged to freely share their lived experiences and perceptions of naturally occurring peer support. They were assured of their right to withdraw from the study at any time without consequence. Prior to audio recording, we would proactively inform the participants and ask for their consent. We informed the participants that only the researchers would have access to the data, which would be used solely for scientific purposes.

Before the recruitment, this study was approved by the Ethics Committee of Shanghai Jiao Tong University, School of Medicine (SJUPN-202126). All participants gave written informed consent, including for the use of anonymised quotes. A compensation of ¥100 was provided to each participant after each interview.

### 2.4 Procedures

LCH and FYJ, both trained in IPA, conducted semi-structured in-depth interviews with lived experience in day-care centres. LCH is expert at qualitative research with years of interview experience and FYJ is her senior student. We designed a semi-structured guide (**Table 1**) and conducted a pre-interview to perfect the questions. We focused on their actual situation, how they interacted with peers in day-care centres and their feelings, changes and difficulties ever since in day-care centres. The guide consisted of 7 questions and probes under the following domains: (a) experience with peers in day-care centres, (b) experience of mutual help with peers, (c) experience of socialisation in day-care centres, (d) decision-making for medical help-seeking and (e) reflections on daily experience. Baseline data were collected from the participants with a printed questionnaire at last.

**Table 1:** Interview questions for lived experience in day-care centres.

Introductory question:
Can you tell me about your experience with your friends at the day-care centre?
Additional questions (to be used if necessary):
1.How do you and your peers interact at the day-care centre?
2.Do you have any close friends there? Can you share any stories about your experiences with them?
3.What do your peers mean to you?
4.What positive/negative influences do they have on you?
5.Are there any limitations to the interaction and mutual support among peers?
6.Do you have any advice on how to improve peer support and make it more effective?

We conducted 14 individual qualitative interviews with day-care centre users. They are from three different districts in Puxi, Shanghai urban areas, including five different day-care centres, where the economic development level is basically representative of the average level of the Shanghai urban area. We conducted interviews between July 2021 to February 2022, predominantly with male participants (n = 10).

Conversations took place in consultation rooms within day-care centres after naptime to ensure privacy and comfort. Participants were encouraged to lead the conversation and share their experiences and insights from their perspectives. All interviews were scheduled in advance with the cooperation of day-care centre managers for better rapport with participants. Interviews were audio recorded and transcribed verbatim in a timely to retain the emotions, including facial expressions and tones precisely. Interviews were done in Chinese and ranged between 35 and 57min, averaging 49.5 min.

### 2.5 Data analysis

We used IPA to dig the underlying meanings of individual experiences as well as participants’ interpretations of those meanings (17). LCH and FYJ together analysed the data referring to Smith and Osborn’s four-stage thematic analysis approach (18). Initially, all 14 interviews were thoroughly read and re-read to enhance familiarity. Texts were coded into meaning units to retain the original sentence’s whole meaning and uncover underlying information to restore authenticity. Then researcher independently selected the most representative sample to group a table of sub-themes which addressed the same issue, further refining them into primary themes. Next, we used the primary theme and sub-theme list to analyse the remaining samples, carefully and flexibly modifying the list. The other researcher proofread and iterated until a consensus was reached. Duplicate or irrelevant codes were removed. The whole process was cyclical and iterative, with delicate deletion of previously subsumed categories resulting in discovery of new elements of the phenomenon. Researcher kept returning to the codes to check if the meanings were correctly included in primary theme list. We iterated until the themes were saturated. The internal logic between the themes generated and the links between the sub-themes contributed to additionally three themes and eight sub-themes:1. reconstructing a social network: an ordinary interpersonal connection, becoming and conducting oneself, proceeding the process of adaptation in the company of peers; 2. balance and multiple roles within the relationship: selective self-exposure, managing proximity and distance; 3. sense of meaning and sense of community: supporting others while empowering oneself, love as expanding consciousness, advocating for the notion of group identity.

## 3 Results

The primary objective of this study was to examine the everyday peer interactions and experiences of mutual help among registered members in day-care centres located in Shanghai, China. A total of 14 participants were involved in our interview-based study conducted between July 2021 and February 2022, as indicated in **Table 2**. Service user participants had a mean age of 40.6 years (SD 6.45 years; range 29-53 years) and were primarily men (10/14, 71.4%) and single (14/14, 100%). Only one participant lives alone; one participant lives with her daughter; others live with their parents. They had an average of 7.64 years (SD 4.01 years; range 1-14 years) experience in day-care centres.

**Table 2:** Characteristics of participants.

Participant/Gender	Diagnosis	Range of age(year)	Marriage	Occupation	Duration of day-care centre(year)	Duration of disease(year)
P1/M	Schizophrenia	41-45	Unmarried	Part-time/Volunteer	13	29
P2/F	Bipolar disorder combined with physical disorders	41-45	Unmarried	Part-time/Volunteer	5	25
P3/M	Schizophrenia	36-40	Unmarried	Unemployed	10	24
P4/M	Schizophrenia	36-40	Unmarried	Unemployed	9	24
P5/M	Schizophrenia	31-35	Unmarried	Unemployed	14	14
P6/M	Schizophrenia	41-45	Unmarried	Unemployed	2	24
P7/M	Epileptic mental disorder	51-55	Unmarried	Unemployed	12	26
P8/F	Schizophrenia	35-40	Unmarried	Unemployed	9	22
P9/M	Obsessive-compulsive disorder	25-30	Unmarried	Part-time/Volunteer	1	9
P10/M	Schizophrenia	36-40	Unmarried	Unemployed	4	16
P11/M	Schizophrenia	41-45	Unmarried	Unemployed	7	15
P12/M	Bipolar disorder	41-45	Divorced	Part-time/Volunteer	5	11
P13/F	Schizophrenia	51-55	Divorced	Retired	10	33
P14/F	Schizophrenia	31-35	Unmarried	Unemployed	6	8

A total of three themes and eight sub-themes were derived from the data analysis, as presented in **Table 3**. The subsequent section of this paper will delve into an analysis of the themes and sub-themes, which will be substantiated by the inclusion of quotations accompanied by pseudonym IDs.

**Table 3:** themes and sub-themes.

theme	sub-theme
Reconstructing a social network	An ordinary interpersonal connection
	Becoming and conducting oneself
	Proceeding the process of adaptation in the company of peers
Balance and multiple roles within the relationship	Selective self-exposure
	Managing proximity and distance
Sense of meaning and sense of community	Supporting others while empowering oneself
	Love as expanding consciousness
	Advocating for the notion of group identity

### 3.1 Reconstructing a social network

By attending the day-care centre, participants were able to interact with new acquaintances and establish new friendships, thus overcoming the loneliness that often accompanies their diagnosis and re-establishing a social network. During social encounters, individuals can really express their genuine selves without concealing their problems. They can enhance adaption by engaging empathic individuals. All participants expressed this theme unanimously. This theme comprises three sub-themes.

#### 3.1.1 An ordinary interpersonal connection

Regarding narratives shared among peers, the primary response from all individuals involved was the presence of a conversational partner. They have the freedom to engage in open discussions on a wide range of subjects, experience a sense of relaxation, and derive pleasure from the experience. They believed they were engaging in typical social interactions, no longer experiencing loneliness, and deriving pleasure from their interactions. They appear to be intentionally preserving this cheerful connection while avoiding discussing each other’s suffering.

*“It’s true that I talk to them more like I would a friend rather than with a specific objective in mind. I’m actually feeling content and at ease during this conversation.” (P4, schizophrenia, male)*

Moreover, participants perceive a sense of shared interests with their peers as a result of their alienation from the external world caused by their disease, which hinders their ability to establish common ground with those outside their circle.

*“I can no longer keep up with my cousins on this topic. They talk among themselves. Here, we have the same conversation. I can’t follow them because a lot of what they discussed is something I haven’t personally experienced. There isn’t a set topic (at the day-care centre). The subject can be anything at all.” (P3, schizophrenia, male)*

#### 3.1.2 Becoming and conducting oneself

Participants, feeling secure and included, were motivated to collectively explore and express themselves. For instance, they engage in the exchange of personal narratives regarding their ailments and cultivate their skills and interests alongside their peers.

*“I would occasionally discuss the precipitating incident, how I came to have this condition, or my own symptomatic behaviours. Sharing them made me feel so much more at ease. I also feel like I’m not fighting this war alone when other people reply with comparable experiences (giggles).” (P4, schizophrenia, male)*

Through the acknowledgement and assistance of their peers, participants are motivated to engage more actively and willingly in exploring their untapped potential.

*“I have changed from being a quiet person to becoming more talkative. Everybody in this room has identified their specialty. Music is my thing, and Z is skilled at crafts. I want to make an effort in that direction today. Since I was little, I have always liked playing the guitar, but I never got the chance to do it. I can make it happen here.” (P5, schizophrenia, male)*

#### 3.1.3 Proceed the process of adaptation in the company of peers

Although discussing symptoms or drug side effects is not a frequent subject among peers, it was occasionally brought up. Several participants conveyed that engaging in discussions about this content aids in their acceptance of their present circumstances and facilitates their adjustment process. Utilizing adaptive measures with unintended consequences as a substitute to reduce the discomfort. While engaging in discussions with peers may not alleviate the symptoms, it can foster self-acceptance and inner tranquillity.

*“Some of them also take medication for OCD, although they claim it doesn’t truly work. I have OCD symptoms. They therefore make the decision to stop using it and attempt to handle things alone. I now take care of it primarily on my own. We all think that changing is difficult. I’ve made the decision to go with the flow because everyone feels that way and it’s unchangeable.” (P3, schizophrenia, male)*

Within certain peer groups, individuals not only engage in emotional contact and provide psychological support to one another, but they also offer aid in managing daily medication and addressing other life matters, thereby assisting their companions in effectively dealing with their own challenges.

*“I quickly tell my friend T to go home and obtain his health insurance card when he runs out of medication while we’re together. Though there are moments when he refuses to take the medication, I go with him to the community health centre to fill his prescription.” (P12, bipolar disorder, male)*

*“I live by myself. Now that I have Z, I can always find her on WeChat when I need her. She was open to hearing what I had to say, sharing my happiness and my sorrows. She helped me get through my credit card crisis several days ago.” (P14, schizophrenia, female)*

### 3.2 Balance and multiple roles within the relationship

The accounts provided by the participants unveiled insights on the concepts of self-disclosure and interpersonal distance. Within this overarching subject, two sub-themes emerged: Selective self-exposure and balancing interpersonal distance.

#### 3.2.1 Selective self-exposure

Participants acquire valuable insights through actively engaging in the process of listening to the narratives of others, while also reciprocating by disclosing their own personal information. Nevertheless, a significant majority of participants shown a hesitancy to engage in conversations pertaining to matters concerning disease and symptoms. There were apprehensions and reservations around the act of self-disclosure, encompassing both the nature of the information shared and the individuals it was disclosed to. Individuals exhibited a propensity to exhibit hesitation when it came to divulging their vulnerabilities, displaying concern regarding the potential reactions of their audience, and harbouring apprehension about potentially disturbing the prevailing atmosphere of ease and comfort.

*“It’s similar to using a filter on your phone, so you can only view the best content presented on the screen and are blind to what else is there. I suppose that’s the inner self’s establishing role. You cannot assist many of your fellow patients because they are uncomfortable discussing these matters with you (Grabs phone and makes motions).” (P12, bipolar disorder, male)*

*“It appears that a lot of individuals find it annoying when you enquire about such things. I mean, they genuinely don’t know how to handle it if you bring up their condition themselves.” (P2, bipolar disorder, female)*

The narratives provided by the participants also indicated that certain individuals would purposefully create space between themselves and their friends while experiencing emotional distress or instability, with the intention of avoiding any disruption to the harmonious atmosphere.

*“I typically visit the Sunshine Soul Park and help others when I’m in a generally positive emotional state. I like to relax and take it easy at home when I’m not feeling well.”(P2, bipolar disorder, female)*

#### 3.2.2 Managing proximity and distance

Various roles and relationships were identified based on the descriptions provided by the participants. The linkages exhibited a notable degree of flexibility and adaptability. These relationships may range from casual acquaintances to confidants with whom individuals share intimate details, and could even extend to soulmates. Certain individuals even formed close and deep connections that resembled familial bonds. The shown flexibility and scalability in their interpersonal relationships exemplified the wide array of connections they established.

*“In reality, we talk a lot outside of here. WeChat voice and video calls are what we utilize the most. We discuss a vast array of subjects and chat about anything. We talk about both good and sad topics. We discuss issues pertaining to our families, events occurring outside, and our thoughts on particular individuals or objects. We regularly talk for two to three hours every day.” (P14, schizophrenia, female)*

As interpersonal connections strengthen, certain individuals may get engaged in the familial dynamics of their peers, taking on the role of facilitating communication between parents and adult children, and even undertaking parental responsibilities on behalf of one another.

*“For instance, T, one of my close friends, suffers from schizophrenia. T’s own mental health was unstable when his father was admitted to the hospital, so he didn’t feel like seeing him there. I tried to persuade him, but I was unable to alter his mind. I visited his father in the hospital by myself in the end. And then his father gave me the responsibility of taking care of his kid while he was getting close to the end of his life.” (P12, bipolar disorder, male)*

*“I even assisted him with personal hygiene, such as bathing, while he was experiencing severe signs of schizophrenia and refused to shower himself.” (P12, bipolar disorder, male)*

The participants’ willingness to create romantic relationships with persons who also had mental health issues revealed the adaptability of their interpersonal dynamics. They often become rational when dealing with potential romantic attractions within peer relationships. Due to internalized stigma and considerations of genetic risks, they consciously distance themselves from such intimate relationships.

*“I know exactly that. I would never pursue a romantic relationship with a female patient, even when I occasionally feel something special for her. Since I’m the only child and have always felt obligated to carry on the family name, I still want to marry a regular person.” (P11, schizophrenia, male)*

*“On a particular occasion, a male co-patient extended an invitation to partake in a meal at a KFC establishment, prompting an immediate sense of unease within me. I experienced an intuitive sensation that he harboured undisclosed intentions; hence I courteously rejected the request.” (P14, schizophrenia, female)*

Based on the provided descriptions of the participants, it appears that peer intimate connections might occasionally induce stress for one party, and the act of excessively revealing personal information may not consistently elicit reciprocal emotions. The interests and requirements of the individuals participating in the contact may not always be congruent.

*“She’s willing to share everything with me. I can feel that she relies on me. even though she’s younger than me. However, there are times when it consumes too much of my time and I feel a little overwhelmed and powerless because I have no better counsel to provide.” (P13, schizophrenia, female)*

*“I once met a female who repeatedly added me back to her WeChat friends list after removing me around seven or eight times. During that time, it was incredibly bothersome and frustrating.” (P14, schizophrenia, female)*

### 3.3 Sense of meaning and sense of community

The participants expressed that they derived a sense of significance from their engagements with their fellow participants. The aforementioned meanings arise from the benefits derived from assisting others, the understanding of love via the practice of empathy and care for others, and the motivation to foster community awareness that transcends individual perspectives.

#### 3.3.1 Supporting others while empowering oneself

Assisting others contributes to personal growth and development. The majority of participants recounted anecdotes about how they employed their skills and past experiences to assist others, resulting in a feeling of achievement and restoration. The provision of good feedback thereafter reinforces their conviction in the act of assisting others.

*“I now teach my friends how to sing every day, and it feels really fulfilling. I intend to continue instructing them for as long as I can. Observing their happiness when they sing makes me feel valuable and purposeful, which makes me happy as well.” (P2, bipolar disorder, female)*

*“My elders gave me advice when I first started working at the bakery as part of my vocational training. Now that I’m a senior, I assist the newcomers in the same way. For instance, I give them advice on how to interact with co-workers without having mental health issues and remind them to cut their nails on time.” (P4, schizophrenia, male)*

#### 3.3.2 Love as expanding consciousness

Through active participation in empathetic exchanges and reciprocal assistance among peers, the individuals inadvertently expand their perspective on the world and reacquaint themselves with the profound significance of “loving others” as a lifestyle choice. They stated that “love constitutes the supreme objective of existence.” The phrase “love” originates from the Chinese Confucian notion of “compassion”, which denotes a benevolent disposition towards all entities in the universe.

The participants consistently affirm that they possess a form of affection known as “empathy”, through which they are able to comprehend the positive or negative emotions of their fellow peers. They demonstrate a sincere effort to accommodate the tempo of others during activities, refraining from hastening or assigning blame.

*“I force myself to slow down when I’m teaching my friends how to make crafts with beads. I am aware that many of them experience difficulties focusing and that the side effects can cause them to move erratically.” (P4, schizophrenia, male)*

*“Are you familiar with Master Hongyi, a well-known social activist and Buddhist master in contemporary China? He defined love as compassion in response to a question. It’s a love and concern for every living thing on the planet, whether or not you know them. It’s inherently human to be concerned. It is as though a Buddha has made his home in my heart. Heart-to-heart conversation without borders. I talk about Buddhism a lot with my friends, and we have a ‘spiritual bond’ that allows us to share happiness. That’s sufficient.” (P12, bipolar disorder, male)*

*“I feel that relationships between people are destined by fate, maybe even from past lives. So, when someone needs and trusts me, I cherish that connection and I’m willing to do everything I can to help. After obtaining my social work certification, I’ve become even more aware of the significance of this.” (P13, schizophrenia, female)*

#### 3.3.3 Advocating for the notion of group identity

While the statements made by the participants indicate a significant amount of social discrimination, they are actively working towards altering society’s prejudices against individuals with mental illness. They are contemplating their own deficiency in assimilating into society and striving to overcome the feeling of disgrace. They are actively challenging themselves to move beyond their familiar and safe environments and fully embrace their true selves as they explore the world beyond.

*“We hear in the news about crimes perpetrated by people suffering from mental illnesses, and we talk about it, reminding ourselves to take our medications on time and keep ourselves out of situations like these. … Break the prejudice in society that mental patients are prone to crime.” (P1, schizophrenia, male)*

This is also evident in the vocational training in day-care centres, where senior members provide reminders and teach job skills to their junior peers to minimize errors. In turn, this act of assistance also enhances the self-esteem of the helper.

*“I feel the need to establish this store as a brand and perform better … We should not permit others to assume that our ailment is the sole cause of our poor health. As a result, I shall endeavour to remind my peers.” (P4, schizophrenia, male)*

Based on the collective experiences of the participants, it has been determined that they possess an insufficient set of abilities necessary for successful reintegration into society. Furthermore, the existing training programs they receive are deemed inadequate in adequately preparing them to navigate the complexities of the external environment.

*“Simply walking into society is insufficient for us; we also require instruction in emotional intelligence, communication skills, maintaining confidentiality, and knowing what to say and what not to say. Everything is well here, but outside they become issues. Therefore, treatment is required. Perhaps the issue is that we are regarded as a unique group here.” (P4, schizophrenia, male)*

A considerable number of individuals involved in this study are the only child of a family, and they have commenced contemplating the retirement prospects for both their own self and their respective parents. Despite the lack of optimism in the current situation, many have not relinquished their hope. Furthermore, several individuals showed a strong inclination towards seeking employment opportunities.

*“Actually, since we are unemployed and unable to support society, we are in a retirement-like situation. We talked about retirement concerns the last time, and we’re all surrounded by elderly individuals. Everyone will ultimately age, so what should we do with ourselves? Should we decide to age in our own homes or rely on social retirement?” (P11, schizophrenia, male)*

*“People’s relationships, in my opinion, are predestined and were intended in a previous existence. I therefore treasure this kind of destiny and am prepared to help as best I can when I am needed and trusted by others.” (P14, schizophrenia, female)*

The participants additionally conveyed their inclination to advocate for their own interests and the interests of their group, surpassing the negative societal perceptions and feelings of disgrace linked to their condition, and actively participating in public awareness campaigns to educate the wider community.

*“I want to work with people to overcome obstacles so that we can all work together and use our individual strengths to reintegrate into society. Obtaining work in order to support oneself. This is really useful.” (P11, schizophrenia, male)*

*“Perhaps I ought to attempt talking to people about my disease and my experience with it.” (P2, bipolar disorder, female)*

*“I am prepared to assume the role of the peer representative and address the audience from the podium.….” (P12, bipolar disorder, male)*

*“I believe I now know how to provide peer assistance. For instance, asking someone straight out about their symptoms—like delusions or tinnitus—might come out as assuming the role of a medical professional and offering them advice from a higher authority. They detest this with a great intensity. Things would be different if you asked, ‘Did anything interesting happen today?’” (P13, schizophrenia, female)*

## 4 Discussion

This study provides the initial analysis of the interaction of naturally occurring peer support observed among individuals in Chinese psychiatric day-care centres. The study’s findings show naturally occurring peer support provided important and meaningful social support for participants. Participants interact with others who have undergone similar conditions, enabling them to rebuild a valuable social network. A multitude of roles and levels of proximity develop among them. Through this reciprocal relationship, individuals are capable of enhancing their own autonomy, raising their sense of self-worth, and igniting a shared sense of identity. The study also underscores the potential fragility and difficulties inherent in this type of connection.

### 4.1 Reconstructing a social network

According to recent research, having peers around can help individuals overcome feelings of isolation and experience the joy of connecting with others (19–21). Peers can provide valuable assistance for recovery from illness, exchange of knowledge, and skill development through peer feedback and encouragement. Cutrona and Suhr (22) identified five categories of social support, including emotional, informational, esteem, social network support, and tangible support, which were all confirmed in the study. Unfortunately, individuals with mental illness often receive less social support than others, despite its importance in addressing life’s challenges. Social support from peers has been linked to adaptive functioning and is recommended as an intervention target to increase hope, reduce self-stigma, and combat loneliness (2, 23). Previous studies have shown that stigma can increase loneliness by leading to avoidance of social situations (24, 25).

Furthermore, the findings revealed peers play a crucial role in facilitating participants to foster a sense of self-esteem and self-worth. There is evidence that perceived support is associated strongly with self-evaluation (26, 27). This may be explained through symbolic interactionism, in which the self-concept is maintained through social relationships. Studies conducted by Corrigan and Rao (28), Taniguchi (29), and Yokoyama et al. (30) have also shown that individuals who share their experiences of mental illness and recovery often report increased self-esteem and overall well-being. The study about recovery stories reflected that self-respect is one of the key resources for mental health recovery (31). Therefore, it was suggested that people with mental illness can enhance their self-concept through using peer support.

### 4.2 Selective self-exposure, managing proximity and distance

According to our research, individuals tend to be selective in sharing personal information with their peers. This may be due to a desire to avoid revealing vulnerabilities, causing concern, or disrupting the soothing nature of their interactions. Our findings align with previous studies that suggest individuals with depression may be hesitant to acknowledge their condition due to the guilt and stigma surrounding it (25). While keeping secrets can provide protection against stigma, it may ultimately result in negative consequences such as social isolation and loneliness (32). In certain western countries, strategic disclosure of personal information has been shown to be beneficial in peer service programs (32–34). However, in societies where mental health is not openly discussed, individuals may perceive more risks than rewards in disclosing their condition (35). Thus, further research on self-disclosure within Chinese culture is needed.

Another finding of this study is the blurred boundaries in participants’ peer relationships. Peer roles are not fixed and can range from casual acquaintances to intimate friends, sometimes resembling familial bonds. However, when peer relationships become imbalanced, one party may feel burdened by either too much closeness or distance. This phenomenon is explained by the famous Chinese sociologist—Xiaotong Fei’s theory of “Chaxugeju: The Differential Mode of Association,” which posits that traditional Chinese patterns of forming interpersonal connections are self-centred (36). Since the aim of peer relationships is often to seek pleasure, the emotional value of peers can naturally affect the pattern of interpersonal connections among them. The misalignment of expectations exerts psychological strain on the other individual, leading to fluctuating levels of proximity between peers. Conversely, improper sacrifice can place an undue burden on one’s peers (37). This finding inspires further exploration into the influence of interpersonal relationship characteristics in Chinese culture on peer support among people with mental illnesses.

### 4.3 Sense of meaning and sense of community

The final finding of this study is that peer support exposes participants to a sense of meaning and love, as well as fostering a sense of collective consciousness. This notion is consistent with the Buddhist philosophy of showing compassion and kindness towards others, as participants expressed genuine care and concern towards one another during their interactions and mutual aid. People with disabilities aspire to live, work, and love in a community where they can make a valuable contribution (38). Peer interactions also foster personal growth and the formation of a collective identity, which is especially crucial for individuals with mental health conditions who encounter societal prejudices. This can also be seen as their own mental health advocacy, which is essential for breaking down stigma and promoting local mental health services. This finding highlights an opportunity to leverage the strengths of peers for the development of recovery-oriented community mental health services in China.

## 5 Future research and recommendation

The state of mental health in China is presently insufficiently developed, characterised by a growing community-based culture. However, there is a significant deficiency in community infrastructure and healthcare personnel. There are advanced community environments where society is proactive and capable of assuming responsibility for caring for mentally disabled populations (39–41). In China, parents assume the primary responsibility for caring for those with mental challenges, and family support plays a significant role. This phenomenon can be partially ascribed to the strong familial ties prevalent in China, as well as the existence of societal barriers that hinder their access to support (13, 42). Hence, it is imperative to establish expansive nursing homes with healthcare professionals and foster more tolerant, flexible workplaces that provide adequate psychoeducation in the future. To ensure the success of treatments led by peers, a more careful co-design approach is necessary.

## 6 Limitations

This study primarily focuses on the daily interaction and mutual assistance among individuals utilising day-care services, without providing a precise definition of peer support recipients and providers. Furthermore, our study exclusively focuses on areas in Shanghai, and the findings may not necessarily be applicable to the entire nation.

## Data availability statement

The original contributions presented in the study are included in the article/supplementary material. Further inquiries can be directed to the corresponding author.

## Ethics statement

The studies involving humans were approved by the Ethics Committee of Shanghai Jiao Tong University, School of Medicine (SJUPN-202126). The studies were conducted in accordance with the local legislation and institutional requirements. The participants provided their written informed consent to participate in this study. Written informed consent was obtained from the individual(s), and minor(s)’ legal guardian/next of kin, for the publication of any potentially identifiable images or data included in this article.

## Author contributions

YF: Conceptualization, Data curation, Formal analysis, Investigation, Methodology, Project administration, Resources, Writing – original draft. XL: Conceptualization, Data curation, Investigation, Project administration, Resources, Writing – review & editing. CL: Conceptualization, Data curation, Formal analysis, Funding acquisition, Investigation, Methodology, Project administration, Resources, Supervision, Writing – review & editing.
